# A Lyapunov-Based Extension to Particle Swarm Dynamics for Continuous Function Optimization

**DOI:** 10.3390/s91209977

**Published:** 2009-12-09

**Authors:** Sayantani Bhattacharya, Amit Konar, Swagatam Das, Sang Yong Han

**Affiliations:** 1 Department of Electronics and Telecommunication Engineering, Jadavpur University, Kolkata 700032, India; E-Mails: bhattacharya.sayantani@gmail.com (S.B.); konaramit@yahoo.co.in (A.K.); swagatamdas19@yahoo.co.in (S.D.); 2 School of Computer Science and Engineering, Chung-Ang University, Seoul 156-756, Korea

**Keywords:** particle swarm dynamics, metaheuristics, continuous function optimization, stability, convergence, lyapunov stability theorem

## Abstract

The paper proposes three alternative extensions to the classical global-best particle swarm optimization dynamics, and compares their relative performance with the standard particle swarm algorithm. The first extension, which readily follows from the well-known Lyapunov's stability theorem, provides a mathematical basis of the particle dynamics with a guaranteed convergence at an optimum. The inclusion of local and global attractors to this dynamics leads to faster convergence speed and better accuracy than the classical one. The second extension augments the velocity adaptation equation by a negative randomly weighted positional term of individual particle, while the third extension considers the negative positional term in place of the inertial term. Computer simulations further reveal that the last two extensions outperform both the classical and the first extension in terms of convergence speed and accuracy.

## 1. Introduction

The concept of particle swarms originated from the simulation of the social behavior commonly observed in animal kingdom and evolved into a very simple but efficient technique for global numerical optimization in recent past. The Particle Swarm Optimization (PSO) [1,2], as it is called now, does not require any gradient information of the function to be optimized, uses only primitive mathematical operators and is conceptually very simple. PSO emulates the swarming behavior of insects, animals herding, birds flocking and fish schooling, where these swarms forage for food in a collaborative manner. PSO also draws inspiration from the boids method of Craig Reynolds and Socio-Cognition [2].

Since its inception, the research on PSO has centered on the improvement of the particle dynamics and the algorithm. Shi and Eberhart incorporated the *inertia factor* [3] in the basic PSO dynamics for faster convergence of the algorithm. Clerc and Kennedy [4] considered in their work an alternative form of PSO dynamics using a parameter called *constriction factor*, and gave a detailed theoretical analysis to determine the value of the parameter. Eberhart and Shi compared the effect of inertia factor and constriction factor on PSO performance [5]. Angeline [6] introduced a form of selection operation in the PSO algorithm, so that the characteristics of good particles are transferred to the less effective members of the swarm to improve their behavior. Suganthan [7] employed a neighborhood operator in the basic particle swarm optimization scheme to study the swarm behavior. Extension of the PSO algorithm to deal with dynamic environment and efficient explorations are undertaken in [8,9]. Ratnaweera *et al.*, while proposing a new model of self-organizing hierarchical PSO [10], ignored the term involving inertia factor from the velocity adaptation rule. Another contribution of this paper is the inclusion of time-varying inertia weight and time-varying acceleration coefficients for better performance of the algorithm.

In [11], a new crossover operator is defined to swap information between two individuals in order to determine their next position on the search landscape. Miranda *et al.* in [12] proposed a mutation operator on the parameters of the PSO dynamics and the position of the neighborhood best particle, so as to enhance the diversity of the particles, thereby increasing the chances of escaping local minima. In [13], the inertia weight is mutated and the particles are relocated when they are too close to each other. A further increase in the diversity of the population has been attained in [14,15] through introduction of a new collision-avoiding mechanism among the particles. Xie *et al.* [16] added negative entropy to the PSO to discourage premature convergence. In [2], a cooperative PSO (CPSO) is implemented to significantly improve the performance of the classical PSO. Hendtlass *et al.* [17] combined Ant Colony Optimization with PSO to determine the neighborhood best of a particle from a list of best positions found so far by all the particles.

Most of existing works on PSO refer to single objective optimization problems. Coello *et al.* first proposed a formulation of multi-objective optimization problem using PSO [18], and later extended it to include a constraint-handling mechanism and a mutation operator [19] to improve the power of exploration of the optimization algorithm. Agrawal, Panigrahi and Tiwari [20] in a recent paper proposed a fuzzy clustering-based PSO algorithm to solve the highly constrained environmental/economic dispatch problem involving conflicting objectives.

There exists an extensive literature on improving the performance of the PSO algorithm. This has been undertaken by two alternative approaches. First, the researchers are keen to improve swarm behavior by selecting the appropriate form of the swarm dynamics. Alternatively, considering a given form of particle dynamics, researchers experimentally, or theoretically, attempted to find the optimal settings of the range of parameters to improve PSO behavior. In this paper, we adopt the first policy to determine a suitable dynamics, and then attempted to empirically determine the optimal parameter settings.

The classical PSO dynamics adapts the velocity of individual particles by considering the inertia of the particle and the position of local and global attractors. The positions of the attractors are also adapted over the iterations of the algorithm. The motion of the particles thus continues until most of the particles converge in the close vicinity of the global optima. In this paper, we consider different versions of the swarm dynamics to study the relative performance of the PSO algorithm both from the point of view of accuracy and convergence time.

The formal basis of our study originates from the well-known Lyapunov's theorem of classical control theory. The Lyapunov's theorem is widely used in nonlinear system analysis to determine the necessary conditions for stability of a dynamical system. In this paper, we indirectly used Lyapunov's stability theorem to determine a dynamics that necessarily converges to an optima of the Lyapunov-like search landscape. The principles of guiding particle dynamics towards the global and local optima, here too, is ensured by adding local and global attractor terms to the modified PSO dynamics. The rationale of selecting a dynamics that converges at one of the optima on a multimodal surface, and the principle of forcing the dynamics to move towards local and global optima together makes it attractive for use in continuous nonlinear optimization.

There are, however, search landscapes that do not possess the necessary characteristics of a Lyapunov surface. This calls for an alternative dynamics, which maintains the motivation of this research but can avoid the restriction on the objective function to necessarily be Lyapunov-like. A look at the dynamics constructed for Lyapunov-like benchmark functions essentially reveals an inclusion of a negative position term in the velocity adaptation rule. This prompted us to realize different variants of the classical PSO dynamics, such as (a) replacement of the inertial term by a negative partial derivative of the Lyapunov-like search landscape, (b) inclusion of a negative particle position in the velocity adaptation rule, (c) replacement of the inertial term by the negative positional term in the dynamics. Computer simulations undertaken on a set of eight benchmark functions reveals that the modifications in the PSO dynamics results in a significant improvement in the PSO algorithm with respect to both convergence speed and accuracy. Note that the extensions developed in this article are primarily meant for fast and accurate optimization of continuous and differentiable functions, as all of them involve first derivatives of the objective function to be used.

The rest of the paper is organized as follows. Section 2 provides a set of formal definitions on Lyapunov stability of nonlinear dynamics. It explains the basis of selection of a dynamics for a given Lyapunov-like objective function. The rationale of speed-up of the proposed swarm algorithm using the selected dynamics is given in this section. Experimental results over several numerical benchmarks are presented in Section 3. Finally the paper is concluded in Section 4.

## 2. Proposed Extensions of the Classical PSO Dynamics

In this section, we briefly outline one typical PSO dynamics, and the PSO algorithm. We next present the possible modifications that we need to undertake in the dynamics to study their relative performance with the classical PSO algorithm.

The global-best (g-best) PSO dynamics for the *j*^th^ particle is given in vector form through Equations 1 and 2:

(1) $${\text{V}}_{j}\left(t+1\right)=\omega {\text{V}}_{j}\left(t\right)+\alpha^{l}\left(t\right)({\text{P}}_{j}^{l}\left(t\right)-{\text{X}}_{j}\left(t\right))+\alpha^{g}\left(t\right)({\text{P}}^{g}\left(t\right)-{\text{X}}_{j}\left(t\right))$$

(2) $${\text{X}}_{j}\left(t+1\right)={\text{X}}_{j}\left(t\right)+{\text{V}}_{j}\left(t+1\right)$$

where:

V*_j_*(*t*) = [v*_j_*_1_(*t*) v*_j_*_2_(*t*)…………… v*_j_*_D_(*t*)] is a *D*-dimensional velocity vector at time *t*,

X*_j_*(*t*) = [x*_j_*_1_(*t*) x*_j_*_2_(*t*)…………… x*_j_*_D_(*t*)] is a *D*-dimensional position vector at time *t*, ${\text{P}}_{j}^{l}\left(t\right)=[{\text{p}}_{j1}^{l}\left(\text{t}\right)\;{\text{p}}_{j2}^{l}\left(\text{t}\right)\ldots \ldots \ldots {\text{p}}_{j\text{D}}^{l}\left(\text{t}\right)]$ is a *D*-dimensional personal (local) best position vector of particle *j*, so far achieved until iteration *t*, ${\text{P}}_{j}^{g}\left(t\right)=[{\text{p}}_{j1}^{g}\left(\text{t}\right)\;{\text{p}}_{j2}^{g}\left(\text{t}\right)\ldots \ldots \ldots {\text{p}}_{j\text{D}}^{g}\left(\text{t}\right)]$ is a *D*-dimensional global best position vector found so far by the entire swarm at iteration *t*.

Empirically, *ω* is a random no. in [0,1], *α^l^*(*t*) and *α^g^*(*t*) are random coefficients in [0, 2] and [0, 4] respectively. Inertia factor *ω* is selected randomly only once in the PSO algorithm, whereas *α^l^*(*t*) and *α^g^*(*t*) are selected randomly in each iteration of the PSO algorithm.

The basic PSO algorithm is presented here for convenience of the readers. The notion of time t is dropped from the algorithm for simplicity.

### PSO-Algorithm

**Begin**

Initialize population;

**While** terminating conditions not reached **do**

**Begin**

**For** *j* = 1 to *N* **do** // *N* = Number of particles//

**Begin**

Evaluate fitness *f*(·)of particle *j*;

**If** $f\left({\text{X}}_{j}\right)<f\left({\text{P}}_{j}^{1}\right)$

$${\text{P}}_{j}^{l}\leftarrow {\text{X}}_{j};$$

**End for;**

$${\text{P}}^{g}\leftarrow \text{Arg}[\text{Min}\left\{f\left({\text{P}}_{1}^{l}\right),f\left({\text{P}}_{2}^{l}\right),\ldots \ldots ‥,f\left({\text{P}}_{N}^{l}\right)\right\}];$$

**For** *j* = 1 to *N* **do**

**Begin**

Adapt ${\text{V}}_{j}\leftarrow \omega {\text{V}}_{j}+\alpha^{l}({\text{P}}_{j}^{l}-{\text{X}}_{j})+\alpha^{g}({\text{P}}^{g}-{\text{X}}_{j});$

Adapt X*_j_* = X*_j_* + V*_j_*;

**End for;**

**End while;**

**End.**

A look at the PSO algorithm reveals that it attempts to determine the optima on a search landscape by allowing several particles (agents) to explore on the surface with an ultimate aim to terminate at the global optima. The terminating condition usually includes an upper limit on the iterations or a lower limit to the unsigned successive difference in the best particle position, or whichever occurs earlier.

In the next sub-section, we would look for a dynamics that has a tendency to move towards optima, which need not essentially be the global optima. This can be attained by identifying a suitable dynamics that ensures asymptotic stability in the vicinity of an optimum over the search landscape. This, of course, needs additional restriction on the surface to satisfy the necessary conditions to be Lyapunov-like [24]. If a suitable dynamics ensuring the convergence to an optimum is identified, we can control the motion of the particles towards the global/local optima by adding global and local attractors in the dynamics as used in the PSO dynamics.

### 2.1. Identifying a Stable Dynamics for a Lyapunov-like Surface

This section begins with a few definitions, available in the standard literature in Nonlinear Control Theory in [21-24], to explain the methodology of determining a stable dynamics for a Lyapunov-like surface. In what follows we shall use the following special notations: ‖X‖ to define the Euclidean norm of a vector.

***S***(*ε*) to denote an *ε*-neighborhood surrounding a point defined by the position-vector X*_e_*. ***S***(*ε*) is basically a set containing all the points in the vector space for which ‖X − X*_e_*‖ ≤ *ε*.

#### Definition 2.1

A point X = X*_e_* is called an *equilibrium state*, if the dynamics of the system which is given by

$$\frac{d\text{X}}{dt}=f\left(\text{X}\left(t\right)\right)$$

becomes zero at X = X*_e_* for any *t*. The equilibrium state is also called equilibrium (stable) point in *D*-dimensional hyperspace, when the state X*_e_* has *D*-components.

#### Definition 2.2

A scalar function *V*(X) is said to be *positive definite* with respect to the point X*_e_* in the region ‖X − X*_e_*‖ ≤ *K*, if *V*(X) > 0 at all points of the region except at X*_e_* where it is zero.

Note that *V*(X) will be called *negative definite* if −*V*(X) is positive definite. A scalar function *V*(X) is said to be *indefinite* in the region ‖X − X*_e_*‖ ≤ *K*, if it assumes both positive and negative values within this region.

#### Definition 2.3

A scalar function *V*(X) is said to be *positive semi-definite* with respect to the point X*_e_* in the region ‖X − X*_e_*‖ ≤ *K*, if its value is positive at all points of the region except at finite number of points including origin where it is zero.

Note that similar as definition 2.2, the scalar function *V*(X) is said to be *negative semi-definite* if –*V*(X) is positive semi-definite.

#### Definition 2.4

A scalar function *V*(X) is called a *Lyapunov surface* with respect to the origin, if it satisfies the three conditions listed below:

- *V*(0) = 0
- *V*(*X*) > 0 for X ≠ 0
- $\frac{\partial V}{\partial x_{i}}$ is a continuous function of *x_i_*, where *x_i_* is the i^th^ component of X

#### Definition 2.5

A dynamics *d*X*/dt* = *f*(X(*t*)) is *asymptotically stable* at the equilibrium point X*_e_*, if

- it is stable in the sense of Lyapunov, *i.e.*, for any neighborhood S(ε) surrounding X*_e_* there is a region S(δ), δ < ε, such that trajectories of the dynamics starting within S(δ) do not leave S(ε) as time t → ∞ and
- the trajectory starting within S(δ) converges to the origin as time t approaches infinity

The sufficient condition for stability of a dynamics can be obtained from the Lyapunov's theorem, presented below.

#### Lyapunov's Stability Theorem [21]

Given a scalar function *V*(X) and some real number ε > 0, such that for all X in the region S(ε) the following conditions hold:

1. *V*(X*_e_*) =0
2. *V*(X) is positive definite.
3. *V*(X) has continuous first partial derivatives with respect to all components of X

Then the equilibrium state X*_e_* of the system *d*X*/dt* = *f*(X(*t*)) is

- *asymptotically stable* if *dV/dt* is negative definite, and
- *asymptotically stable* in the large if *dV/dt* is negative definite, and in addition, *V*(X) → ∞ as ‖X − X*_e_*‖ → *∞*

##### Example 1

Let $V(\text{X})=x_{1}^{2}+x_{2}^{2}$ be a Lyapunov energy function for the given dynamics $\frac{dx_{1}}{dt}=-x_{1}$ and $\frac{dx_{2}}{dt}=-x_{2}$ with the equilibrium point X*_e_* = [0,0]. Then:

$$\begin{array}{c} \frac{dV}{dt}=\frac{\partial V}{\partial x_{1}}\frac{dx_{1}}{dt}+\frac{\partial V}{\partial x_{2}}\frac{dx_{2}}{dt}\\ =2x_{1}\left(-x_{1}\right)+2x_{2}\left(-x_{2}\right)\\ =-2\left(x_{1}^{2}+x_{2}^{2}\right)<0 \end{array}$$

*i.e.*, negative definite.

Here, *V*(X) satisfies the first two criterions indicated in the theorem, and the partial derivatives ∂*V*/∂*x*_1_ and ∂*V*/∂*x*_2_ are also continuous functions of *x*_1_ and *x*_2_. Consequently, the asymptotic stability of the dynamics is ensured as *dV/dt* is found to be negative definite for all points except at *x* = 0. Further, as ‖X‖ → ∞, *V*(X) also approaches infinity. Therefore, the asymptotic stability of the dynamics in the large is also ascertained.

The condition for asymptotic stability, as indicated in Theorem1, can be applied to the particle swarm optimization to ensure stability of the dynamics, thereby reducing the convergence time of the algorithm.

When all the three underlying conditions of a Lyapunov function, indicated in Definition 2.4 are supported by the objective function, we would be interested to determine the dynamics that satisfies the necessary conditions for asymptotic stability of the dynamics. It follows from Lyapunov's Theorem that the asymptotic stability of an equilibrium state guided by the dynamics *dx_i_/dt* is ascertained if:

(3) $$\frac{df}{dt}=\sum_{i=1}^{D}\frac{\partial f}{\partial x_{i}}\frac{dx_{i}}{dt}<0$$

The inequality (3) essentially holds when:

(4) $$\frac{dx_{i}}{dt}=-\frac{\partial f}{\partial x_{i}}$$

It is indeed important to note that the condition (4) holds for the *i*-th dimension of a particle roaming over the Lyapunov-like surface for 1 ≤ *i*≤ *D*.

##### Example 2

In this example, we would like to determine a stable dynamics for a Lyapunov-like objective function. Consider for instance the Griewank function in D-dimension:

$$f(\text{X})=\frac{1}{4000}\sum_{i=1}^{D}x_{i}^{2}-\prod_{i=1}^{D}\cos\left(\frac{x_{i}}{\sqrt{i}}\right)+1$$

In order to have asymptotic stability of the dynamics, we set:

$$\frac{dx_{i}}{dt}=-\frac{\partial f}{\partial x_{i}}=\frac{x_{i}}{2000}+\frac{1}{\sqrt{i}}\sin\left(\frac{x_{i}}{\sqrt{i}}\right)\left[\prod_{j=1,j\neq i}^{D}\cos\left(\frac{x_{j}}{\sqrt{j}}\right)\right]$$

It is also apparent to note that the given function *f*(X) satisfies the three necessary conditions of a Lyapunov function. Now, if we replace the term involving inertia factor by the obtained value of *dx_i_/dt* in the PSO dynamics, then the PSO is expected to converge very quickly as the necessary condition for asymptotic stability has been satisfied while deriving the dynamics:

$$\frac{dx_{i}}{dt}=\frac{x_{i}}{2000}+\frac{1}{\sqrt{i}}\sin\left(\frac{x_{i}}{\sqrt{i}}\right)\left[\prod_{j=1,j\neq i}^{D}\cos\left(\frac{x_{j}}{\sqrt{j}}\right)\right]$$

Table 1 provides a list of eight typical benchmark functions along with the derived expressions for *dx_i_/dt* that ensures the asymptotic stability of the derived dynamics over the Lyapunov-like objective function.

We now define Lyapunov-based PSO dynamics (LyPSO) by adding the local and global attractor terms of classical PSO to the derived expression for asymptotically stable Lyapunov dynamics, given in Equations (5) and (6):

(5) $$V_{j}(t+1)=-\omega .\vec{\nabla }f(X)+\alpha^{l}\left(t\right)(P_{j}^{l}\left(t\right)-X_{j}\left(t\right))+\alpha^{g}\left(t\right)(P_{j}^{g}\left(t\right)-X_{j}\left(t\right))$$

(6) $${\text{X}}_{j}\left(t+1\right)={\text{X}}_{j}\left(t\right)+{\text{V}}_{j}\left(t+1\right)$$

Note that ∇⃗*f* = [∂*f/*∂*x*_1_,…, ∂*f/*∂*x_D_*] denotes the gradient of the scalar function *f* to be optimized. The first term in the right hand side of Equation (5) ensures motion of the particle towards minima, while the second and third term controls the motion towards local and global optima respectively. It is apparent from Table 1 that *dx_i_/dt* obtained for different Lyapunov-like surfaces include a factor of (−*x_i_*). The condition for *dx_i_/dt* = −*ωx_i_* is tabulated for all the eight benchmark functions in Table 2. Consequently, instead of computing *dx_i_/dt* by the approach stated earlier, we can simply add a term –*ωx_i_* to the *i*^th^ component of the updated velocity in the classical PSO. The resulting dynamics then looks like Equations (7) and (8):

(7) $$V_{j}\left(t+1\right)=-\omega .{\text{X}}_{j,i}\left(t\right)+\omega .V_{j,i}\left(t\right)+\alpha^{l}\left(t\right)({\text{P}}_{j}^{l}\left(t\right)-X_{j}\left(t\right))+\alpha^{g}\left(t\right)(P_{i}^{g}\left(t\right)-X_{j}\left(t\right))$$

(8) $${\text{X}}_{j}\left(t+1\right)={\text{X}}_{j}\left(t\right)+{\text{V}}_{j}\left(t+1\right)$$

The dynamics given by Equations (7) and (8) is referred to as Position-based PSO (PPSO).

For the sake of completeness of our study, we consider a third category of the dynamics, where the inertial term is dropped from the PSO dynamics, indicated in Equations (9) and (10). The modified dynamics, called Steepest-PSO (SPSO) for its fast convergence (vide Section 3), is formally given below:

(9) $$V_{j}\left(t+1\right)=-\omega .{\text{X}}_{j}\left(t\right)+\alpha^{l}\left(t\right)({\text{P}}_{j}^{l}\left(t\right)-X_{j}\left(t\right))+\alpha^{g}\left(t\right)(P_{i}^{g}\left(t\right)-X_{j}\left(t\right))$$

(10) $${\text{X}}_{j}\left(t+1\right)={\text{X}}_{j}\left(t\right)+{\text{V}}_{j}\left(t+1\right)$$

In the next section, we would justify the reason for accuracy and speed-up of PPSO and SPSO over classical PSO.

### 2.2. The Rationale of Speed-up of the PPSO and SPSO Dynamics over Classical PSO

To compare the relative performance in speed-up and convergence of the proposed algorithms, we study the stability behavior of the proposed PPSO and SPSO dynamics, in absence of the local and the global attractors. This is performed by solving the first order difference equations. The condition for asymptotic stability and the location of the stable point can be ascertained from the solution of the dynamics. Theorems 1 to 2 provide interesting results, indicating asymptotic stability of the SPSO and PPSO dynamics to the origin irrespective of the search landscape, whereas Theorem 3 indicates asymptotic stability of the classical PSO to a stable point, which need not essentially be the origin. The rate at which the particle position approaches the origin further indicates that the speed of convergence of the SPSO algorithm far exceeds that of PPSO, while the speed of PPSO algorithms beats classical PSO.

#### Theorem 1

*The dynamics of the j^th^ particle in the i^th^ dimension given by*

(11) $$v_{j,i}\left(t+1\right)=-\omega x_{j,i}\left(t\right)$$

*has a stable point at the origin, when ω ≤ 1*.

##### Proof

Let *E* be an extended difference operator, such that

$$E(x_{j,i}(t))=x_{j,i}(t)+\Delta x_{j,i}(t)=(1+\Delta )x_{j,i}(t)=x_{j,i}(t+1)$$

Now extending the concept of derivatives to the discrete time domain, Equation (11) now can be written as

(12) $$\begin{array}{c} \frac{x_{j,i}\left(t+1\right)-x_{j,i}\left(t\right)}{\left(t+1\right)-\left(t\right)}=-\omega x_{j,i}\left(t\right)\\ \Rightarrow x_{j,i}\left(t+1\right)-x_{j,i}\left(t\right)=-\omega x_{j,i}\left(t\right) \end{array}$$

Replacing *x_j,i_* (*t*+1) by E(*x_j,i_* (*t*)) in (12) we obtain:

$$\begin{array}{l} Ex_{j,i}\left(t\right)-\left(1-\omega \right)x_{j,i}\left(t\right)=0\\ \Rightarrow \left(E-1+\omega \right)x_{j,i}\left(t\right)=0\\ ∴E=1-\omega \end{array}$$

Consequently, the solution of the dynamics (11) is given by

(13) $$x_{j,i}\left(t\right)=A{\left(1-\omega \right)}^{t}$$

where A is a constant. The expression (13) indicates that for *ω* < 1, *x_j,i_* (*t*)→0 when *t*→∞.Therefore, the dynamics is asymptotically stable at the origin for *ω* < 1. When *ω* = 1, *x_j,i_* (*t*) = 0 at all time *t*. Hence, the theorem follows.

#### Theorem 2

*The dynamics of the j^th^ particle in the i^th^ dimension given by*

(14) $$v_{j,i}\left(t+1\right)=\omega v_{j,i}\left(t\right)-\omega x_{j,i}\left(t\right)$$

*Is asymptotically stable with a stable point at the origin, when ω < 1*.

##### Proof

We can rewrite Equation (14) as

(15) $$\frac{x_{j,i}\left(t+1\right)-x_{j,i}\left(t\right)}{\left(t+1\right)-\left(t\right)}=-\omega x_{j,i}\left(t\right)+\omega x_{j,i}\left(t\right)-\omega x_{j,i}\left(t-1\right)$$

Replacing *x_j,i_* (*t* + 1) by *E*(*x_j,i_*(*t*)) and *x_j,i_* (*t* − 1) by *E*^−1^(*x_j,i_*(*t*)) in Equation (15), we obtain

$$\begin{array}{l} \Rightarrow Ex_{j,i}\left(t\right)-x_{j,i}\left(t\right)+\omega E^{-1}x_{j,i}\left(t\right)=0\\ \Rightarrow \left(E^{2}-1+\omega \right)x_{j,i}\left(t\right)=0\\ \Rightarrow E^{2}=1-\omega \\ \Rightarrow E=\pm \sqrt{1-\omega } \end{array}$$

So, the solution of the dynamics (16) is given by:

(16) $$x_{j,i}\left(t\right)=A{\left(\sqrt{1-\omega }\right)}^{t}-B{\left(\sqrt{1-\omega }\right)}^{t}$$

where A and B are constants. It is apparent from expression (16) that *x_j,i_*(*t*) asymptotically converges to the origin for *ω* < 1. Therefore, the dynamics is asymptotically stable with a stable point at the origin for *ω* < 1. When *ω* = 1, *x_i_*(*t*) = 0 for all *t*. This proves the Theorem.

#### Theorem 3

*The dynamics of j^th^ particle in the i^th^ dimension given by:*

(17) $$v_{j,i}\left(t+1\right)=\omega v_{j,i}\left(t\right)$$

*is asymptotically stable and it converges to a stable point, which need not essentially be zero*.

##### Proof

We can rewrite Equation (17) as:

(18) $$\frac{x_{j,i}\left(t+1\right)-x_{j,i}\left(t\right)}{\left(t+1\right)-\left(t\right)}=\omega \left[\frac{x_{j,i}\left(t\right)-x_{j,i}\left(t-1\right)}{t-\left(t-1\right)}\right]$$

Let *E* be an extended difference operator, such that *E^n^x_j,i_*(*t*) = *x_j,i_*(*t* + *n*), and *E^−n^x_j,i_*(*t*) = *x_j,i_*(*t* − *n*) for any positive integer *n*. Consequently, Equation (18) is transformed to:

$$\begin{array}{l} \;Ex_{j,i}\left(t\right)-\left(1+\omega \right)x_{j,i}\left(t\right)+\omega E^{-1}x_{j,i}\left(t\right)=0,\\ \Rightarrow E^{2}x_{j,i}\left(t\right)-\left(1+\omega \right)Ex_{j,i}\left(t\right)+\omega x_{j,i}\left(t\right)=0,\\ \Rightarrow \left(E^{2}-\left(1+\omega \right)E+\omega \right)x_{j,i}\left(t\right)=0,\\ \Rightarrow \left(E^{2}-E-\omega E+\omega \right)=0,\\ \Rightarrow \left(E-\omega \right)\left(E-1\right)=0.\\ ∴E=\omega ,1. \end{array}$$

So, the solution of the dynamics (17) is given by:

(19) $$\begin{array}{l} x_{j,i}\left(t\right)=A{\left(1\right)}^{t}+B\omega^{t}\\ \Rightarrow x_{j,i}\left(t\right)=A+B\omega^{t}, \end{array}$$

whereAand Bare constants. It is apparent from expression (21) that xj,i(t) asymptotically converges to Aas time tapproaches infinity. SinceAis not zero unconditionally, therefore the statement of the theorem follows.

**Figure 1.**
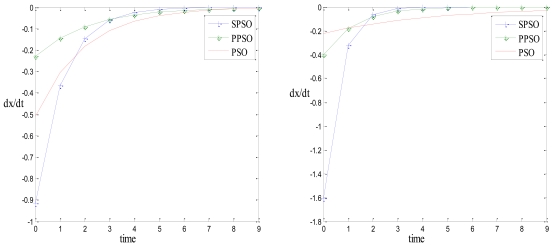
Variation of *dx_i_/dt* with respect to time: (a) for *ω* = 0.6. (b) for *ω* = 0.8.

Table 3 provides the results of computation of dxi/dt for SPSO, PPSO and classical PSO. The computations in the table are performed from Equations 13, 16 and 19 respectively. Figure 1 shows the variation of dxi/dtwith respect to time forω=0.6 andω=0.8. It is apparent from the graphs that in the absence of local and global attractors, the dynamics of SPSO converges faster than that of PPSO, which further converges faster than the classical PSO.

## 3. Computer Simulations and Experimental Results

### 3.1. Benchmarks

In order to study the performance of the proposed three alternative PSO dynamics, we used eight well-known benchmark functions as listed in Table 1. All the functions listed here have global minima at the origin except the Rosenbrock function. The performance of the three proposed dynamics for these eight functions is compared with that of classical PSO.

### 3.2. Parametric Range and Error Criterion

Early methods of performance evaluations for evolutionary algorithms were restricted to symmetric initializations. In recent time, researchers prefer asymmetric initialization [25]. We here used the asymmetric initialization method to evaluate the performance of proposed three dynamics along with the classical PSO. In Table 4, we provide the initialization range of the objective function variables, the position of theoretical optima and the error criterion used to terminate the algorithm. Different error criterions were used for different benchmark functions.

### 3.3. Simulation Strategies

Parameter selection of the PSO dynamics also is a crucial issue for speed-up and accuracy of the PSO algorithm. For a given benchmark function, we initially took wider range of the PSO dynamics parameters: *α^g^*(*t*), *α^l^*(*t*) and *ω*. The initial ranges selected in our simulation were *α^g^*(*t*) in [0, 4], *α^l^*(*t*) in [0, 2] and *ω* < 1. Several hundred runs of the PSO programs with random parameter settings in the above ranges confirm that for a specific function, the best choice of parameters are restrictive as indicated in Table 5.

The following observations readily follow from Table 5.

#### Observation 1

For each benchmark function, the parameter set of the dynamics including *α^g^* (*t*), *α^1^* (*t*) and ω for LyPSO has a relatively restricted range than those of PPSO and SPSO.

#### Observation 2

The parameter sets for most of the benchmark functions for the LyPSO dynamics have a common range as listed below: *α^g^* (*t*) in [0.1999, 0.5999] and *α^1^* (*t*) in [0.0001, 0.001]. The parameter sets for most of the benchmark functions for the PPSO and SPSO dynamics have a common range as listed below: *α^g^* (*t*) in [0.199, 0.999], *α^1^* (*t*) in [0.0001, 0.01] and *ω* in [0.3, 0.6]. Moreover, the size of the population is taken as 40 and a maximum of 5,000 iterations were taken for 30-dimensional particles.

### 3.4. Experimental Results from the Simulations

**Figure 2.**
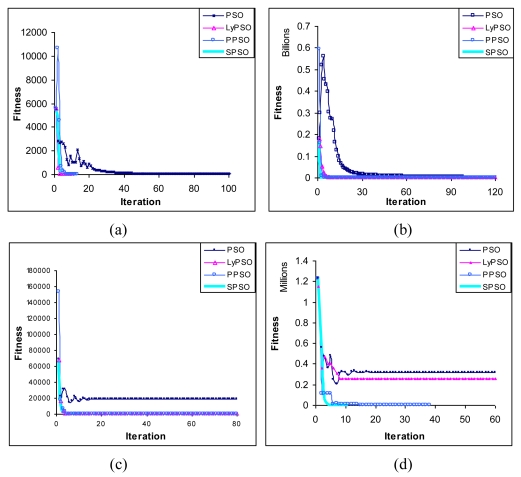

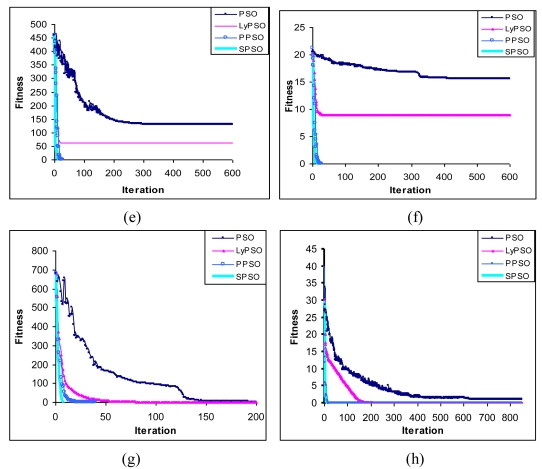
Progress towards the optima: (a) Sphere function. (b) Rosenbrock's function. (c) Step function. (d) Schwefel's Problem 1.2. (e) Rastrigin's function. (f) Ackley's function. (g) Griewank's function. (h) Salomon's function.

The relative comparison of the convergence time of the three algorithms with respect to classical PSO are given in Figure 2a–h. It is observed from these figures that SPSO always outperforms PPSO in convergence time and accuracy. It is further revealed from these graphs that PPSO yields better performance in accuracy and convergence time with respect to both classical PSO and LyPSO. The performance of the four algorithms is summarized with a ‘≤’ operator, where, x ≤ y indicates that performance of y is better than or equal to that of x. Relative performance:

Classical PSO≤LyPSO≤PPSO≤SPSO

Table 6 provides the mean error and standard deviation for the globally best particle obtained by execution of the PPSO, SPSO, LyPSO and classical PSO over eight benchmark functions. The error was obtained by taking the Euclidean distance between the theoretical optima and the position of the best-fit particle for a given program run. The mean error designates the average of errors over 50 independent runs. In order to make the comparison fair enough, runs of all the algorithms were let start from the same initial population. The variance denotes the second moment of the errors with respect to the mean error. It is clear from Table 6 that for mean error for the SPSO algorithm is comparable but less than that obtained by PPSO algorithm, and the mean error obtained by the PPSO algorithm is insignificantly less than that of LyPSO algorithm. Further, the mean error obtained by the LyPSO algorithm is less in comparison to that of the classical PSO algorithm. This confirms that the SPSO algorithm outperforms the PPSO and LyPSO and definitely the classical PSO algorithm from the point of view of accuracy in solution. The speed-up of the SPSO algorithm has already been demonstrated in graphs *vide* Figures 2a–2h.

Table 7 shows results of unpaired *t*-tests between the best and second best algorithms in each case (standard error of difference of the two means, 95% confidence interval of this difference, the *t* value, and the two-tailed *P* value). For all cases, sample size = 50 and degrees of freedom = 98. It is interesting to see from Tables 6 and 7 that one or more of the proposed PSO methods can always beat the classical PSO in a statistically significant way.

Our experimental results suggest that for multi-modal problems having the fitness landscape punctuated with multiple local optima, the SPSO dynamics is the most preferable choice. However, for uni-modal functions, LyPSO and SPSO are nearly equivalent in terms of their final accuracy and convergence speed.

**Figure 3.**
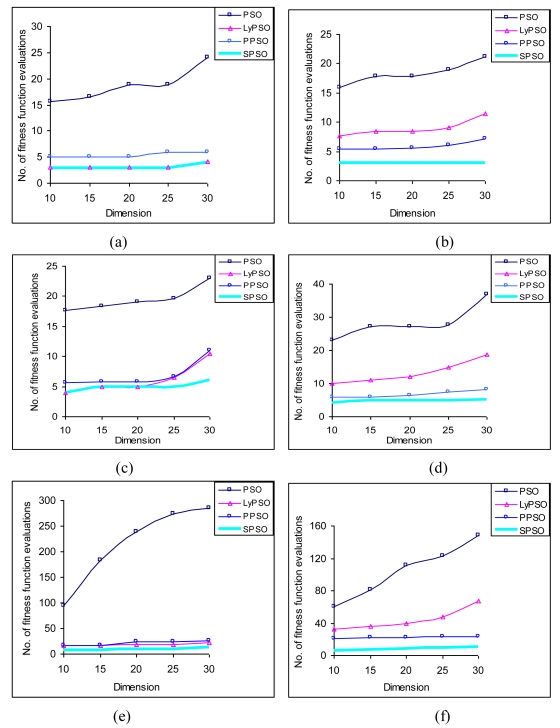

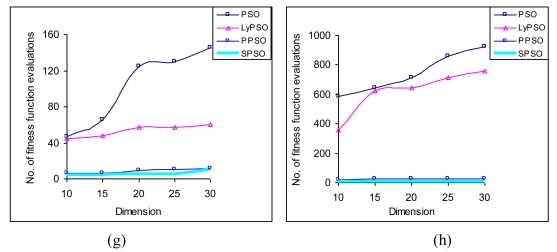
Variation in number of fitness function evaluations with function dimension: (a) Sphere function. (b) Rosenbrock's function. (c) Step function. (d) Schwefel's Problem 1.2. (e) Rastrigin's function. (f) Ackley's function. (g) Griewank's function. (h) Salomon's function.

In order to compare the scalability of the proposed PSO-variants against the growth of dimensionality of the search space, we need to plot the no. of fitness function evaluations with dimension of the search landscape. The results shown in figures are average over 50 independent runs of the PSO program. It is clear from Figure 3 that the number of Fitness Function evaluations for PPSO and SPSO do not increase significantly in comparison to that of LyPSO and classical PSO algorithms.

The PPSO, SPSO, LyPSO and PSO algorithms have been executed on eight benchmark functions, and for each algorithm the average of the convergence time for 50 independent runs to meet the error limit for individual function as specified in Table 6 is recorded in Table 8. It is clear from this Table that the mean convergence time of the SPSO is less than that of PPSO. The mean convergence time of PPSO is less than that of LyPSO, and the latter is less than the mean convergence time of classical PSO. The above phenomena is true for all benchmark functions except the sphere, where the LyPSO and SPSO gives identical results because of same functional form in the SPSO and LyPSO dynamics.

## 4. Conclusions

Classical g-best PSO has a proven impact in optimization of multi-modal nonlinear objective functions. However, for many nonlinear continuous multi-modal functions, where partial derivatives with respect to objective function variables exist, classical g-best PSO is not very efficient as it does not utilize gradient information of the search landscape. The paper bridges the gap between gradient-free and gradient-based optimization algorithm. It does not truly utilize gradient information of the search space, but it requires the background information that the gradient of the surface exists. When the prerequisite knowledge about the search space is known, we extend the classical g-best PSO algorithm by the principles outlined in the paper.

Three alternative approaches to improve the speed of convergence of the PSO dynamics over continuous fitness landscapes is discussed in the paper. The first approach attempted to replace the inertial term in the dynamics by a factor that ensures asymptotic stability of the PSO dynamics. Construction of such dynamics presumes the characteristics of the surface being Lyapunov-like. This, however, is not a very restrictive assumption as many multi-modal surfaces support the conditions for Lyapunov function. On the contrary, the Lyapunov-based extension, even without local and global attractors, has a natural tendency to move towards optima on the surface. The convergence of the algorithm to local and global optima, however, is controlled by the presence of attractors in the PSO dynamics.

The second alternative approach to make the PSO smarter was derived from the Lyapunov-based formulation, just by noting that the Lyapunov-based dynamics includes a factor of negatively weighted position of the particle. Incorporation of this new term to the existing velocity adaptation rule classical PSO gives birth to the second alternative form of the extended PSO dynamics. The resulting dynamics has been found to have asymptotic stability for a selective range of ω < 1, *i.e.*, same as in classical PSO. The third extension lies in replacement of the inertial term by the negative position of the particle itself. A random factor is attached to this term to maintain explorative power of the PSO dynamics to avoid its premature convergence. Computer simulations undertaken ensure that the third alternative form of extended PSO dynamics results in significant improvement in convergence time and accuracy compared to the results obtained by the first and second attempt. However, all three approaches outperform the classical PSO dynamics from the point of view of the convergence time and accuracy.

Future research efforts will focus on the extensions of Lyapunov-based dynamics of gbest PSO for handling non-continuous multi-modal functions. The particle dynamics will be combined with an estimate of the gradient of the function, instead of using any analytical expression of the partial derivatives of the objective function. Also the Lyapunov-based particle dynamics will be examined in context to non-linearly constrained optimization problems, where only a portion of the search space will be used to generate feasible optimal solutions.

## Figures and Tables

**Table 1.** The derived dynamics for the selected benchmark functions.

Function name	Functional form *f*(**X**(*t*))	*dx_i_/dt*
Sphere Function	$f(\text{X})=\sum_{i=1}^{D}x_{i}^{2}$	−2*x_i_*
Rosenbrock's Function	$f(\text{X})=\sum_{i=1}^{D-1}\left[100{\left(x_{i+1}-x_{i}^{2}\right)}^{2}+{\left(x_{i}-1\right)}^{2}\right]$	$-\left(400x_{i}^{2}+2\right)x_{i}+2\left(1+200x_{i}x_{i+1}\right)$
Step Function	$f(\text{X})=\sum_{i=1}^{D}{\left(\left|x_{i}+0.5\right|\right)}^{2}$	−2|*x_i_* + 0.5|
Schwefel's Problem 1.2	$f(\text{X})=\sum_{i=1}^{D}{\left(\sum_{j=1}^{i}x_{j}\right)}^{2}$	$-2x_{i}\left(\sum_{j=1}^{i-1}x_{j}\right)$
Rastrigin's Function	$f(\text{X})=\sum_{i=1}^{D}\left[x_{i}^{2}-10\cos(2\pi x_{i})+10\right]$	−2*x_i_* −20*π* sin (2*πx_i_*)
Ackley's Function	$\begin{array}{l} f(\text{X})=-20\exp(-0.2\sqrt{\frac{1}{D}\sum_{i=1}^{D}x_{i}^{2})}\\ -\exp(\frac{1}{D}\sum_{i=1}^{D}\cos2\pi x_{i})+20+e \end{array}$	$\begin{array}{l} -\frac{4}{D}\left(\exp\left(-0.2\sqrt{\frac{1}{D}\sum_{i=1}^{D}x_{i}^{2}}\right)\right)\frac{x_{i}}{\sqrt{\frac{1}{D}\sum_{i=1}^{D}x_{i}^{2}}}\\ -\frac{2\pi }{D}\sin2\pi x_{i}\left[\exp\left(\frac{1}{D}\sum_{i=1}^{D}\cos2\pi x_{i}\right)\right] \end{array}$
Griewank's Function	$f(\text{X})=\frac{1}{4000}\sum_{i=1}^{D}x_{i}^{2}-{∐}_{i=1}^{D}\cos\left(\frac{x_{i}}{\sqrt{i}}\right)+1$	$-\frac{x_{i}}{2000}-\frac{1}{\sqrt{i}}\sin\left(\frac{x_{i}}{\sqrt{i}}\right)\left[\prod_{j=1,j\neq i}^{D}\cos\left(\frac{x_{j}}{\sqrt{j}}\right)\right]$
Salomon's function	$f\left(\text{X}\right)=-\cos\;\left(2\pi \sqrt{\sum_{i=1}^{D}x_{i}^{2}}\right)+0.1\sqrt{\sum_{i=1}^{D}x_{i}^{2}}+1$	$\begin{array}{l} -2\pi x_{i}{\left(\sum_{i=1}^{D}x_{i}^{2}\right)}^{-\frac{3}{2}}\sin\left(2\pi \sqrt{\sum_{i=1}^{D}x_{i}^{2}}\right)\\ -0.1x_{i}{\left(\sum_{i=1}^{D}x_{i}^{2}+1\right)}^{-\frac{3}{2}} \end{array}$

**Table 2.** Reduced form of *dx_i_/dt*.

**Function name**	**Reduced form of**$\frac{dx_{i}}{dt}$	**Condition for reduction**
Sphere Function	−2*x_i_*	Unconditional
Rosenbrock's Function	$-x_{i}\left(400x_{i}^{2}+2-400x_{i+1}\right)$	When $x_{i}x_{i+1}>>-\frac{1}{200}$
Step Function	−2|*x_i_*|	When *x_i_* ≫0.5
Schwefel's Problem 1.2	$-2x_{i}\left(\sum_{j=1}^{i-1}x_{j}\right)$	Unconditional
Rastrigin's Function	−*x_i_*(2+4*π*^2^)	When *x_i_* is very small.
Ackley's Function	$-x_{i}\left(\begin{array}{l} \frac{4}{D}\left(\exp\left(-0.2\sqrt{\frac{1}{D}\sum_{i=1}^{D}x_{i}^{2}}\right)\right)\frac{x_{i}}{\sqrt{\frac{1}{D}\sum_{i=1}^{D}x_{i}^{2}}}\\ +\frac{2\pi }{D}\left[\exp\left(\frac{1}{D}\right)\right] \end{array}\right)$	When *x_i_* is very small.
Griewank's Function	$-x_{i}\left(\frac{1}{2000}+\frac{1}{i}\right)$	When *x_i_* is very small
Salomon's function	$-x_{i}\left(\begin{array}{l} 2\pi {\left(\sum_{i=1}^{D}x_{i}^{2}\right)}^{-\frac{3}{2}}\sin\left(2\pi \sqrt{\sum_{i=1}^{D}x_{i}^{2}}\right)\\ +0.1{\left(\sum_{i=1}^{D}x_{i}^{2}+1\right)}^{-\frac{3}{2}} \end{array}\right)$	Unconditional

**Table 3.** *dx_i_/dt* for SPSO, PPSO and classical PSO.

**Dynamics**	*dx_i_/dt*
**SPSO**	*A*(1−ω)^t^ log _e_(1−ω)
**PPSO**	$\frac{\left(A-B\right)}{2}{\left(1-\omega \right)}^{t/2}\log_{e}\left(\sqrt{1-\omega }\right)$
**Classical PSOB**	*Bω^t^* log _e_*ω*

**Table 4.** Parametric range of benchmark functions.

**Function Name**	**Dimension**	**Initialization Range**	**Theoretical Optima**	**Error Criterion**
Sphere Function	30	[50, 100]	[0,0……,0]	0.01
Rosenbrock'sFunction	30	[15, 30]	[1,1……,1]	0.001
Step Function	30	[50, 100]	[0,0……,0]	0.01
Schwefel's Problem 1.2	30	[50, 100]	[0,0……,0]	0.001
Rastrigin's Function	30	[2.56, 5.12]	[0,0……,0]	0.1
Ackley's Function	30	[15, 32]	[0,0……,0]	0.01
Griewank's Function	30	[300, 600]	[0,0……,0]	0.001
Salomon's function	30	[50, 100]	[0,0……,0]	0.001

**Table 5.** Range of optimal values of *α^g^* (*t*), *α^1^* (*t*) and *ω* of LyPSO, PPSO and SPSO.

**Function Name**	**Parameters for PSO Algorithm**					
	
	*α^g^* (*t*), *α^1^* (*t*) and *ω* of LyPSO			*α^g^* (*t*), *α^1^* (*t*) and *ω* of PPSO/SPSO		
	*α^g^* (*t*)	*α^1^* (*t*)	*ω*	*α^g^* (*t*)	*α^1^* (*t*)	*ω*
Sphere Function	0.9999–1.9999	0.0001–0.001	0.5–0.7	0.1999–1.9	0.0001–0.01	0.4–0.7
Rosenbrock's Function	0.1999–0.3999	0.001–0.003	10^−12^–10^−11^	0.001–0.999	0.001–0.009	0.3–0.6
Step Function	0.9999–1.9999	0.0001–0.001	0.5–0.7	0.199–0.999	0.001–0.01	0.3–0.7
Schwefel's Problem 1.2	0.599–0.799	0.001–0.003	10^−12^–10^−9^	0.199–0.999	0.001–0.01	0.4–0.7
Rastrigin's Function	0.2999–0.5999	0.0001–0.0005	0.0001–0.0009	0.2999–0.5999	0.0001–0.0005	0.3–0.6
Ackley's Function	0.1–0.2	0.7–0.8	0.001	Random	Random	0.3–0.7
Griewank's Function	Random	Random	56–60	Random	Random	0.3–0.7
Salomon's function	0.1999–0.5999	0.0001–0.0005	5000	0.1999–0.5999	0.0001–0.0005	0.3–0.6

**Table 6.** Mean error and standard deviation over the benchmarks.

**Function Name**	**Dimension**	**Classical PSO**	**LyPSO**	**PPSO**	**SPSO**

		**Mean Error (Standard Deviation)**	**Mean Error (Standard Deviation)**	**Mean Error (Standard Deviation)**	**Mean Error (Standard Deviation)**
Sphere Function	30	2.04e+00 (1.08e+00)	4.3e−03 (7.94e−04)	1.32e−02 (4.00e−03)	4.3e−03 (7.94e−04)
Rosenbrock's Function	30	7.77e+00 (0.77e+00)	1.58e+00 (2.07e−01)	9.99e−01 (9.4e−04)	9.94e−01 (2.7e−03)
Step Function	30	2.45e+01 (1.56e+00)	5.00e−01 (1.00e−03)	3.42e−01 (8.62e−02)	2.46 e−01 (1.01e−01)
Schwefel's Problem 1.2	30	4.2.4e+01 (6.35e+00)	1.89e+01 (1.86e+00)	2.7e−03 (1.3e−03)	1.90e−03 (1.20e−03)
Rastrigin's Function	30	2.97e+00 (1.9e−01)	1.48e+00 (8.06e−02)	2.70e−03 (8.27e−04)	2.79e−04 (6.82e***−***05)
Ackley's Function	30	7.03e+00 (6.22e+00)	2.76e+00 (3.85e−01)	1.70e−03 (6.20e−04)	1.74e−04 (4.95e−05)
Griewank's Function	30	1.73e+00 (1.13e+00)	9.93e−01 (3.00e−03)	5.17e−02 (1.81e−02)	1.58e−02 (3.50e−03)
Salomon's function	30	1.14e+01 (9.66e+00)	4.44e+00 (1.25e+00)	2.43e−01 (1.57e−01)	6.03e−04 (1.65e−04)

**Table 7.** Results of unpaired t-tests on the data of Table 6.

**Function**	**Std. Err**	***t***	**95% Conf. Intvl**	**Two-tailed *P***	**Significance**
Sphere Function	0.000	21040.9635	–0.16340 to –0.16337	<0.0001	Extremely significant
Rosenbrock's Function	0.000	13.3484	–0.00585820 to –0.00434179	<0.0001	Extremely significant
Step Function	0.019	5.1050	–0.1329012 to –0.0584988	<0.0001	Extremely significant
Schwefel's Problem 1.2	0.000	3.1974	–0.0012965 to –0.0003035	0.0019	Very statistically significant
Rastrigin's Function	0.000	206.2488	–0.002444193 to –0.002397606	<0.0001	Extremely Significant
Ackley's Function	0.000	191.1514	0.0016651516 to 0.0017000883	<0.0001	Extremely Significant
Griewank's Function	0.007	4.8989	–0.0504426 to –0.0213574	<0.0001	Extremely significant
Salomon's function	0.022	10.9511	–0.286844994 to –0.198834365	<0.0001	Extremely significant

**Table 8.** Mean convergence time of the benchmarks over 30-dimensions.

**Function Name**	**Dimension**	**Mean Convergence Time (in seconds)**			

		**Classical PSO**	**LyPSO**	**PPSO**	**SPSO**
Sphere Function	30	24.0	4.8	6.0	4.8
Rosenbrock'sFunction	30	21.1	11.5	7.2	5.9
Step Function	30	23.0	10.5	11.0	6.6
Schwefel's Problem 1.2	30	36.8	18.6	8.3	4.3
Rastrigin's Function	30	563.5	23.0	26.0	9.0
Ackley's Function	30	148.9	67.2	23.2	10.9
Griewank's Function	30	172.1	60.5	11.5	8.0
Salomon's function	30	925.9	758.7	28.4	17.2
